# Effects of Transcutaneous Electrical Nerve Stimulation on Pain and Menstrual Symptoms in Primary Dysmenorrhea: A Placebo‐Controlled Study

**DOI:** 10.1111/jmwh.70138

**Published:** 2026-06-07

**Authors:** Esra Ünal

**Affiliations:** ^1^ Faculty of Health Sciences Osmaniye Korkut Ata University Osmaniye Turkey

**Keywords:** menstrual pain, nonpharmacological pain management, primary dysmenorrhea, placebo effect, transcutaneous electrical nerve stimulation, women's health

## Abstract

**Introduction:**

Primary dysmenorrhea, with a prevalence of 45% to 95%, is a common gynecological condition that significantly affects women's daily functioning and quality of life.

**Methods:**

This randomized, single‐masked study compared the effects of transcutaneous electrical nerve stimulation (TENS) on pain intensity and menstrual symptoms in women with primary dysmenorrhea using a 3‐arm design including TENS (n = 31), placebo (n = 31), and no‐intervention control (n = 31) groups. TENS was administered in two 20‐minute sessions on the same day during the first 3 days of menstruation. Pain intensity was measured using the Visual Analog Scale (VAS), and menstrual symptoms were assessed with the Daily Menstrual Symptom Rating (DMSR) scale. Between‐group differences in postintervention outcomes were analyzed using analysis of covariance, adjusting for baseline values. The trial was prospectively registered at ClinicalTrials.gov (NCT06741059).

**Results:**

After baseline adjustment, significant between‐group differences were observed in pain intensity and menstrual symptoms. Adjusted posttest VAS scores were lowest in the TENS group, followed by the placebo group and the control group (*P* < .001), with a large effect size (partial η^2^ = 0.428). Adjusted posttest DMSR scores were significantly lower in the TENS group compared with the control group (*P* < .01) with a large effect size (partial η^2^ = 0.160); however, no significant difference was observed between the TENS and placebo groups. Pain scores in the placebo group were also lower than those in the control group, indicating a notable placebo effect.

**Discussion:**

TENS represents a safe, accessible, and patient‐centered option for short‐term menstrual pain management beyond placebo effects in primary dysmenorrhea.

## INTRODUCTION

Primary dysmenorrhea is characterized by lower abdominal pain occurring during menstruation in the absence of any underlying pelvic pathology and represents one of the most common gynecological conditions among women of reproductive age.^1^ With a reported prevalence ranging from 45% to 95%, primary dysmenorrhea not only causes pain but also leads to limitations in daily activities, school and work absenteeism, reduced social functioning, and a significant decline in quality of life.^2^, ^3^

The pathophysiology of primary dysmenorrhea is primarily attributed to increased prostaglandin synthesis, resulting in uterine hyperactivity, reduced uterine blood flow, and myometrial ischemia.^4^, ^5^ Pain arising from these mechanisms is commonly managed with nonsteroidal anti‐inflammatory drugs and hormonal therapies. However, the limited effectiveness of these pharmacologic treatments in some individuals, their potential adverse effects with long‐term use, and intolerance among certain women have increased interest in nonpharmacological and complementary approaches.^6^, ^7^

In recent years, increasing attention has been directed toward nonpharmacological interventions for managing primary dysmenorrhea, including exercise, heat therapy, massage, herbal applications, and electrical stimulation.^2^, ^7^, ^8^, ^9^ Among these, transcutaneous electrical nerve stimulation (TENS) has been highlighted for its potential to modulate pain transmission and central pain mechanisms.^6^, ^10^, ^11^ Although randomized controlled trials and systematic reviews suggest that TENS may reduce pain intensity,^12^, ^13^ methodological limitations—most notably the frequent absence of placebo (sham) control group—complicate the interpretation of its true physiologic effects and the differentiation from expectancy‐related placebo responses.^13^, ^14^ In this context, placebo‐controlled, 3‐arm study designs are of critical importance for elucidating the true effects of TENS in primary dysmenorrhea. The inclusion of a placebo group allows not only the differentiation between intervention effects and natural disease progression but also the control of expectancy effects, thereby enhancing the reliability of study findings.

**QUICK POINTS**

✦ Nonpharmacologic interventions play an important role in the holistic management of primary dysmenorrhea, particularly for women who have limited benefit from or prefer to avoid pharmacologic treatments.
✦ Transcutaneous electrical nerve stimulation (TENS) applied during menstruation was associated with clinically meaningful reductions in menstrual pain compared with both placebo and no‐intervention conditions and reduced overall menstrual symptom burden compared with no intervention.
✦ TENS represents a safe, accessible, and patient‐centered nonpharmacologic option that can be integrated into routine nursing, midwifery, and women's health care practice for short‐term management of primary dysmenorrhea.

The aim of this study was to evaluate the effects of TENS on pain and menstrual symptoms in women with primary dysmenorrhea by comparing TENS with placebo and control groups. The unique strength of this study lies in its comparison of TENS not only with a control group but also with a placebo application, as well as in its use of statistical methods that control for baseline measurements (analysis of covariance [ANCOVA]) to determine the true group effect. In this respect, the present study aims to provide methodologically robust and clinically meaningful evidence to address existing gaps in the literature on the use of TENS in the management of primary dysmenorrhea.

## METHODS

### Study Design

The present study was designed as a randomized, single‐masked study with 2 control groups (no‐intervention control group and placebo treatment). The flow of participants through the study is presented in accordance with the Consolidated Standards of Reporting Trials (CONSORT) guidelines (Figure 1). The CONSORT checklist was used to guide the reporting of the study (Appendix S1). The study protocol was approved by an institutional scientific research and publication ethics committee (decision number: 2024/01‐16, date: May 23, 2024) and prospectively registered at ClinicalTrials.gov (identifier: NCT06741059). In addition, institutional permission was obtained from the administration of a public dormitory where the study was conducted.

### Recruitment Procedure

All eligible participants were informed about the study objectives and procedures, and written informed consent was obtained from all participants prior to participation. Participation in the study was voluntary, and no financial incentives or compensation were provided. The study was conducted between December 20, 2024, and August 10, 2025, among women residing in a public dormitory in Turkey. Public dormitories are state‐operated student residences that provide accommodations for female university students in Turkey and university students from different academic departments. Recruitment was conducted through an announcement in the dormitory stating that an intervention study would be conducted for students experiencing menstrual pain. Students who reported experiencing menstrual pain and who were willing to participate voluntarily presented themselves for screening. Participants were considered to have presumed primary dysmenorrhea based on self‐reported menstrual pain and the absence of any known or previously diagnosed gynecological pathology associated with secondary dysmenorrhea.

### Inclusion and Exclusion Criteria

Inclusion criteria were: (1) being aged 18 years or older, (2) experiencing primary dysmenorrhea without any diagnosed pelvic pathology, (3) having a baseline Visual Analog Scale (VAS) menstrual pain score of 4 or higher, (4) being within the first 3 days of menstruation at the time of intervention, and (5) willingness to participate and provide written informed consent. Postintervention VAS scores were not subject to a minimum threshold because reductions in pain intensity were expected following the intervention. Exclusion criteria included diagnosis of secondary dysmenorrhea or any gynecological pathology, presence of skin lesions or dermatological conditions at electrode application sites, use of a pacemaker or implanted cardiac device, neurological disorders affecting pain perception, prior experience with TENS therapy, and use of analgesic medication on the day of data collection prior to the intervention. Participants who reported analgesic use during the screening process were excluded before randomization.

### Sample Size Calculation

An a priori sample size calculation was performed using G*Power 3.1 software. The calculation was based on the effect size reported in a previous randomized controlled study in which the effects of TENS were evaluated across experimental, placebo, and control groups. In that study, ANCOVA demonstrated a large effect size for pain outcomes (partial η^2^ [ηp^2^] = 0.274).^15^ Accordingly, the power analysis was conducted using ANCOVA with fixed effects, considering 3 groups and one covariate (baseline pain score). With a significance level of 0.05 and a statistical power of 95%, the minimum required total sample size was calculated as 74 participants. Considering potential data loss during the study process, the sample size was increased, and a total of 93 participants were included, with 31 participants allocated to each group.

### Randomization and Allocation Concealment

Participants (N = 93) were randomly assigned to one of 3 groups—TENS (n = 31), placebo (n = 31), or control (n = 31)—using block randomization with randomly varying block sizes to ensure balanced group allocation (Figure 1). The random allocation sequence was generated using the Random.org online random number generation tool by an independent researcher not involved in participant recruitment, intervention provision, data collection, outcome assessment, or statistical analysis. Allocation concealment was ensured using coded group labels (A, B, and C), which were kept confidential until completion of data collection.

### Masking

The study was conducted as a single‐masked trial. Participants in the TENS and placebo groups were anonymized to their group assignment. To maintain masking in the placebo group, participants were connected to the TENS device in the same manner as those in the active intervention group, without provision of electrical stimulation. Indicator lights were activated to mimic an active intervention. Because participants in the control group did not receive device application, anonymization was not feasible for this group. Participants were informed that electrical stimulation devices may or may not produce a tingling sensation and that the presence or absence of sensation would not indicate group assignment. Data collection, outcome assessment, and statistical analyses were conducted using anonymized group codes, which were revealed only after completion of all analyses. Success of anonymizing was not formally assessed.

### Interventions

Participants in the TENS group received TENS using a portable TENS device (VZN Medical Equipment, Turkey) operated in TENS mode. The intervention was administered during the first 3 days of menstruation, when menstrual pain was expected to be present. Following completion of baseline questionnaire assessments, TENS was applied in 2 sessions on the same day, each lasting 20 minutes, with an approximately 2‐hour interval between sessions. The protocol was designed to reflect practical, short‐term pain management during the peak period of dysmenorrheic pain. Previous randomized controlled trials and systematic reviews indicate that TENS is most effective when applied during acute menstrual pain and that repeated sessions within the same day may help sustain analgesic effects during peak symptom periods.^6^, ^13^, ^14^ A total of 4 self‐adhesive surface electrodes (2 channels) were used for each participant. Electrode placement was individualized according to the participant's primary pain location. In participants reporting predominantly lower abdominal pain, electrodes were applied to the lower abdominal (suprapubic/inguinal) region, whereas in participants reporting predominantly low back pain, electrodes were applied to the sacral‐lumbar region. Only one anatomical region was stimulated per participant; simultaneous stimulation of the abdominal and sacral‐lumbar regions was avoided to prevent overlapping stimulation and potential confounding effects. TENS was provided using a predefined high‐frequency conventional TENS program (∼100 Hz) with biphasic symmetrical square‐wave pulses. Stimulation parameters were kept constant throughout the intervention. Stimulation intensity was gradually increased from zero at the beginning of each session and adjusted to a strong but comfortable sensory level, defined as the maximum intensity tolerated by the participant without pain or discomfort. Selection of the stimulation program and verification of device use were performed in consultation with a licensed physiotherapist. Participants in the placebo group underwent the same procedural setup, including electrode placement, session duration, and timing; however, the device was not activated to provide electrical stimulation. The device emitted a visual indicator light but produced no electrical output. Participants in the control group did not receive any device application and completed the same assessment schedule, including a 2‐hour waiting period between baseline and posttest assessments.

### Data Collection

Data were collected from participants who met the inclusion criteria and voluntarily agreed to participate in the study. All data collection procedures were carried out by an independent researcher anonymized to group allocation. Baseline assessments were completed prior to the intervention, and posttest assessments were conducted after completion of the intervention procedures on the same day. The same outcome measures were administered at both time points, with the exception of the descriptive information form, which was completed only at baseline.

The descriptive information form was developed by the researchers based on a review of the relevant literature. The form consisted of 16 items assessing participants’ sociodemographic and menstrual characteristics, including age, education level, employment status, income level, body mass index, age at menarche, menstrual cycle characteristics, duration of dysmenorrheic pain, pain management behaviors, and family history of dysmenorrhea.

Pain intensity was assessed using the VAS. The VAS consists of a 10‐cm horizontal line, with the left end point indicating “no pain” (0) and the right end point indicating “the most severe pain imaginable” (10). Participants were asked to mark the point that best represented their perceived pain intensity. The VAS has been widely used in both clinical practice and research settings to evaluate pain severity and treatment effectiveness. In this study, a baseline VAS score of 4 or higher was required for inclusion.^16^

Menstrual symptoms were assessed using the Daily Menstrual Symptom Rating (DMSR) scale, which was developed in 1979 to evaluate the type and intensity of symptoms experienced during menstruation.^17^ The Turkish validity and reliability study of the scale was conducted by Oskay et al.^18^ The scale consists of 17 items, each rated on a scale from 0 (no symptom) to 5 (very severe symptom). The items assess a range of emotional, behavioral, and physical symptoms associated with menstruation. The total score ranges from 0 to 85, with higher scores indicating greater symptom severity. In the original validation study, the Cronbach's α coefficient was reported as 0.88.^18^ Internal consistency was assessed using baseline data. In the present study, the internal consistency reliability of the DMSR was found to be 0.806.

### Statistical Analysis

Statistical analyses were performed using IBM SPSS Statistics for Windows, version 25.0 (IBM Corp., Armonk, NY). Descriptive statistics were presented as mean and SD for continuous variables and as frequency and percentage for categorical variables. Baseline differences among the TENS, placebo, and control groups were examined using the Kruskal‐Wallis test for continuous variables and χ^2^ tests for categorical variables. To evaluate the effects of the intervention on pain intensity and menstrual symptoms, ANCOVA was conducted. ANCOVA was used to compare posttest scores among groups while statistically controlling for baseline (pretest) values. Group assignment (TENS, placebo, control) was entered as the independent variable, posttest scores were used as the dependent variables, and corresponding pretest scores were included as covariates. Separate ANCOVA models were conducted for the VAS scores and the DMSR total scores. The assumptions of ANCOVA were evaluated prior to analysis, including normality of residuals, linearity between covariates and dependent variables, and homogeneity of regression slopes. When statistically significant between‐group differences were detected in adjusted posttest means, Bonferroni‐adjusted post hoc comparisons were conducted to identify specific group differences. Effect sizes were calculated using ηp^2^, with values of 0.01, 0.06, and 0.14 interpreted as small, medium, and large effects, respectively. A 2‐tailed *P* value of less than .05 was considered statistically significant for all analyses.

## RESULTS

The baseline sociodemographic and menstrual characteristics of the participants are summarized in Table 1. Overall, the TENS, placebo, and control groups showed similar baseline characteristics in terms of age, age at menarche, body mass index, smoking and alcohol use, menstrual cycle characteristics, pain management behaviors, and family history of dysmenorrhea (*P* > .05). Although statistically significant differences were observed in income level and the impact of dysmenorrhea on social functioning (*P* < .05), the overall baseline profiles of the groups were considered similar. Baseline VAS and DMSR scores were similar across the study groups (Tables 2 and 3), indicating that the groups were comparable prior to the intervention.

**Table 1:** Baseline Sociodemographic and Menstrual Characteristics of Participants by Study Group

Characteristic	TENS (n = 31)	Placebo (n = 31)	Control (n = 31)	*P*
**Income level, n (%)**				.01
Less than expenses	11 (35.5)	2 (6.5)	14 (45.2)	
Equal to expenses	16 (51.6)	26 (83.9)	15 (48.4)	
Greater than expenses	4 (12.9)	3 (9.7)	2 (6.5)	
**BMI, n (%)**				.42
Underweight	5 (16.1)	2 (6.5)	5 (16.1)	
Normal weight	23 (74.2)	22 (71.0)	21 (67.7)	
Overweight	3 (9.7)	7 (22.6)	5 (16.1)	
**Smoking status, n (%)**				.23
Yes	5 (16.1)	1 (3.2)	4 (12.9)	
No	26 (83.9)	30 (96.8)	27 (87.1)	
**Alcohol use status, n (%)**				.77
Yes	1 (3.2)	1 (3.2)	2 (6.5)	
No	30 (96.8)	30 (96.8)	29 (93.5)	
**Duration of dysmenorrheic pain, n (%)**				.32
Only first day	2 (6.5)	4 (12.9)	2 (6.5)	
First 2 days	13 (41.9)	14 (45.2)	21 (67.7)	
First 3 days	14 (45.2)	11 (35.5)	8 (25.8)	
≥4 days	2 (6.5)	2 (6.5)	0 (0)	
**Absence from school due to dysmenorrhea, n (%)**				.18
Yes	12 (38.7)	6 (19.4)	7 (22.6)	
No	13 (41.9)	11 (35.5)	9 (29.0)	
Sometimes	6 (19.4)	14 (45.2)	15 (48.4)	
**Impact of dysmenorrhea on social functioning, n (%)**				.05
Yes	16 (51.6)	14 (45.2)	20 (64.5)	
No	0 (0)	4 (12.9)	0 (0)	
Sometimes	15 (48.4)	13 (41.9)	11 (35.5)	
**Impact of dysmenorrhea on interpersonal communication, n (%)**				.22
Yes	17 (54.8)	19 (61.3)	21 (67.7)	
No	3 (9.7)	6 (19.4)	1 (3.2)	
Sometimes	11 (35.5)	6 (19.4)	9 (29.0)	
**Use of pain management strategies for dysmenorrhea, n (%)**				.36
Yes	30 (96.8)	31 (100)	29 (93.5)	
No	1 (3.2)	0 (0)	2 (6.5)	
**Painkiller use, n (%)**				.57
Yes	20 (64.5)	17 (54.8)	16 (51.6)	
No	11 (35.5)	14 (45.2)	15 (48.4)	
**Resting by lying down, n (%)**				.94
Yes	23 (74.2)	24 (77.4)	24 (77.4)	
No	8 (25.8)	7 (22.6)	7 (22.6)	
**Applying heat, n (%)**				.67
Yes	17 (54.8)	20 (64.5)	20 (64.5)	
No	14 (45.2)	11 (35.5)	11 (35.5)	
**Walking, n (%)**				.58
Yes	6 (19.4)	6 (19.4)	9 (29.0)	
No	25 (80.6)	25 (80.6)	22 (71.0)	
**Massage, n (%)**				.50
Yes	8 (25.8)	11 (35.5)	7 (22.6)	
No	23 (74.2)	20 (64.5)	24 (77.4)	
**Consumption of herbal teas, n (%)**				.20
Yes	11 (35.5)	18 (58.1)	15 (48.4)	
No	20 (64.5)	13 (41.9)	16 (51.6)	
**Family history of dysmenorrhea in mother and sister, n (%)**				.68
Yes	21 (67.7)	23 (74.2)	24 (77.4)	
No	10 (32.3)	8 (25.8)	7 (22.6)	
**Age, mean (SD), y**	21.13 (2.32)	21.94 (3.52)	21.39 (1.82)	.72
**Age at menarche, mean (SD), y**	13.65 (1.33)	13.45 (1.23)	14 (2.1)	.66
**Intermenstrual interval, mean (SD), d**	28.52 (4.12)	28.39 (1.91)	26.87 (4.3)	.33
**Menstrual duration, mean (SD), d**	5.9 (1.83)	5.71 (1.16)	5.61 (1.28)	.82

Abbreviations: BMI, body mass index; TENS, transcutaneous electrical nerve stimulation.

*P* values for categorical variables were calculated using the χ^2^ test; *P* values for continuous variables were calculated using the Kruskal‐Wallis test.

**Table 2:** Comparison of Posttest‐Adjusted DMSR Total Scores Among the TENS, Placebo, and Control Groups

Group	Pretest Mean (SD)	Posttest Mean (SD)	Adjusted Mean (SE)	*P*	Post Hoc	Partial η^2^
**TENS (n = 31)^a^**	51.32 (12.08)	31.10 (11.32)	30.36 (2.02)	<.001	a < c	0.160
**Placebo (n = 31)^b^**	51.55 (11.89)	37.55 (15.98)	36.64 (2.02)			
**Control (n = 31)^c^**	48.19 (11.00)	40.48 (15.16)	42.14 (2.03)			

Abbreviations: ANCOVA, analysis of covariance; DMSR, Daily Menstrual Symptom Rating; TENS, transcutaneous electrical nerve stimulation.

^a,b,c^Bonferroni post hoc test; different superscript letters indicate statistically significant differences between groups.

*P* represents the overall group effect based on ANCOVA controlling for pretest scores.

**Table 3:** Comparison of Posttest‐Adjusted VAS Scores Among the TENS, Placebo, and Control Groups

Group	Pretest Mean (SD)	Posttest Mean (SD)	Adjusted Mean (SE)	*P*	Post Hoc	Partial η^2^
**TENS (n = 31)^a^**	6.58 (1.77)	2.97 (2.01)	3.02 (0.25)	<.001	a < b < c	0.428
**Placebo (n = 31)^b^**	6.71 (1.75)	4.52 (1.96)	4.47 (0.25)			
**Control (n = 31)^c^**	6.65 (1.52)	5.94 (1.71)	5.94 (0.25)			

Abbreviations: ANCOVA, analysis of covariance; TENS, transcutaneous electrical nerve stimulation; VAS, Visual Analog Scale.

^a,b,c^Bonferroni post hoc test; different superscript letters indicate statistically significant differences between groups.

*P* represents the overall group effect based on ANCOVA controlling for pretest scores.

As shown in Table 2, a significant difference was observed among the TENS, placebo, and control groups in posttest DMSR total scores after adjusting for baseline values (ANCOVA, *P* < .001). The adjusted posttest mean DMSR score was lowest in the TENS group compared with the control group, indicating a greater reduction in menstrual symptoms in participants who received TENS. Bonferroni post hoc analysis revealed a statistically significant difference between the TENS and control groups, whereas no significant differences were found between the TENS and placebo groups or between the placebo and control groups. The effect size for the group effect was large (ηp^2^ = 0.160).

As presented in Table 3, a statistically significant difference was observed among the TENS, placebo, and control groups in posttest VAS scores after adjustment for baseline values (ANCOVA, *P* < .001). The adjusted posttest mean VAS score was lowest in the TENS group, followed by the placebo group, and highest in the control group. Bonferroni post hoc analysis demonstrated significant differences between all groups (TENS < placebo < control). The group effect was large, with ηp^2^ value of 0.428. Despite minor within‐group variability, baseline‐adjusted between‐group comparisons clearly favored the TENS group.

Figure 2 illustrates the between‐group differences in adjusted posttest VAS scores, demonstrating a clear gradient across the TENS, placebo, and control groups. Although Table 3 presents the statistical comparisons, Figure 1 also provides a visual representation of the adjusted VAS scores across the study groups.

**Figure 1:**
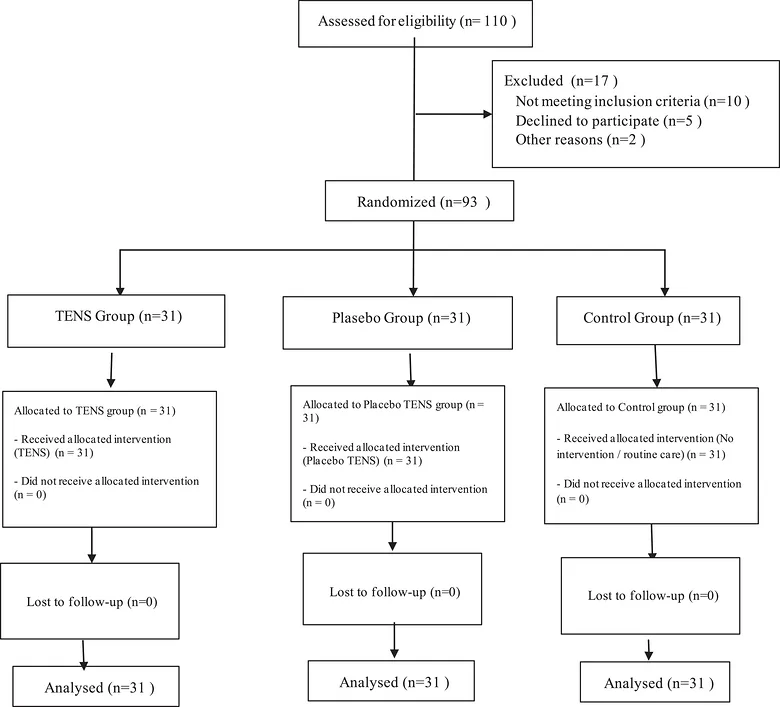
CONSORT 2010 Flow Diagram of the Study Abbreviations: CONSORT, Consolidated Standards of Reporting Trials; TENS, transcutaneous electrical nerve stimulation.

**Figure 2:**
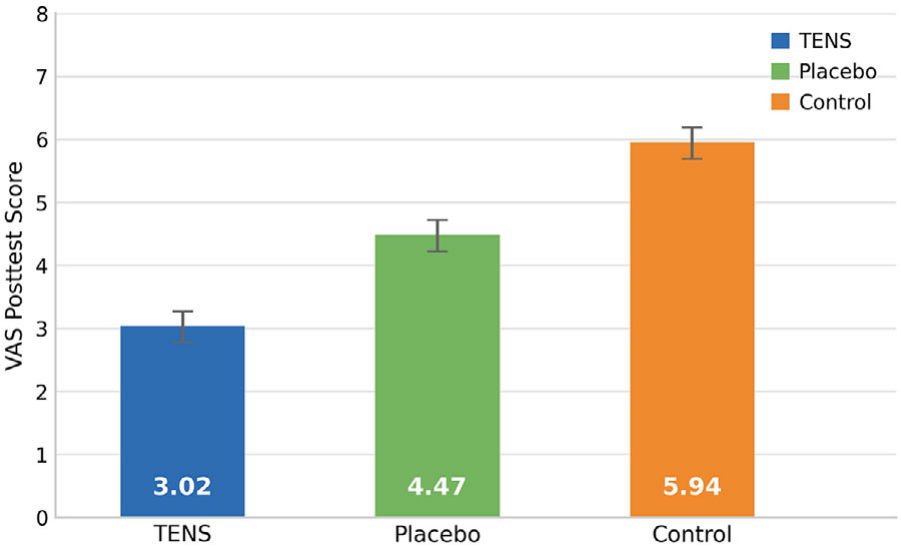
Adjusted Posttest Mean VAS Scores After Controlling for Pretest VAS Scores Error bars represent the SE. Abbreviations: TENS, transcutaneous electrical nerve stimulation; VAS, Visual Analog Scale.

## DISCUSSION

The present study examined the effects of TENS on pain intensity and menstrual symptoms in women with primary dysmenorrhea using a 3‐arm design that included both placebo and control conditions. Baseline characteristics were largely comparable across groups, supporting the internal validity of the findings.

### Effect of TENS on Menstrual Symptoms (DMSR)

After controlling for baseline values, a significant between‐group difference was observed in posttest DMSR total scores, with the lowest adjusted scores in the TENS group compared with the control group. This finding indicates that TENS may reduce not only pain but also the broader constellation of menstrual‐related symptoms, such as fatigue, mood changes, and functional discomfort, which collectively contribute to the overall burden of dysmenorrhea. The observed large effect size (ηp^2^ = 0.160) suggests a clinically meaningful impact and is consistent with previous randomized trials and systematic reviews reporting improvements in dysmenorrhea‐related symptoms following electrical stimulation‐based interventions.^11^, ^13^, ^19^ However, no statistically significant difference was detected between the TENS and placebo groups in DMSR scores. Although the difference between the TENS and placebo groups did not reach statistical significance, the TENS group demonstrated lower adjusted symptom scores compared with the control group after adjustment for baseline values, suggesting that the intervention effect became more evident when compared with the untreated control group. This finding aligns with accumulating evidence highlighting the substantial role of expectancy, contextual, and psychosocial factors in shaping subjective symptom reporting in nonpharmacological interventions.^7^, ^14^ Menstrual symptom perception, which encompasses emotional, cognitive, and somatic components, may be particularly susceptible to placebo responses, thereby diminishing the observable contrast between active and sham interventions when multidimensional symptom scales are used. Similar challenges have been noted in prior dysmenorrhea studies employing composite symptom outcomes, underscoring the importance of placebo‐controlled designs when interpreting intervention effects beyond pain alone.^2^

### Effect of TENS on Pain Intensity (VAS)

In contrast to menstrual symptom outcomes, pain intensity assessed by VAS demonstrated clear and graded differences among all 3 groups, with the lowest adjusted posttest scores in the TENS group, followed by the placebo group, and the highest scores in the control group. The large effect size observed for pain reduction (ηp^2^ = 0.428) provides strong evidence for the analgesic efficacy of TENS in primary dysmenorrhea. These findings are in close agreement with randomized controlled trials and recent systematic reviews reporting significant pain relief associated with TENS application compared with both sham and no‐intervention conditions.^6^, ^11^, ^13^, ^20^ These findings further support the role of electrical stimulation in reducing pain signals and improving pain outcomes in women with primary dysmenorrhea. The significant difference observed between the placebo and control groups further emphasizes the role of placebo mechanisms in pain modulation. Nevertheless, the superior outcome in the TENS group indicates an additional physiologic analgesic effect beyond expectancy alone. This pattern is consistent with the proposed mechanisms of TENS, including modulation of nociceptive transmission through spinal gate‐control mechanisms and activation of endogenous opioid pathways at both spinal and supraspinal levels.^1^, ^6^ This finding highlights the importance of baseline‐adjusted analyses in dysmenorrhea research, wherein symptom severity may vary substantially across menstrual cycles and between individuals. Similar methodological considerations have been emphasized in recent dysmenorrhea trials to avoid misinterpretation of raw pre‐post comparisons.^7^, ^21^

From a practical standpoint, the findings support the use of TENS as a flexible and patient‐centered intervention that can be readily integrated into routine nursing, midwifery, and women's health care settings for the management of primary dysmenorrhea. Given its nonpharmacological nature, ease of application, and favorable safety profile, TENS may represent a valuable complementary option for women who prefer to avoid or cannot tolerate pharmacologic treatments.

### Strengths and Limitations

The present study has several important strengths that enhance the methodological rigor and interpretability of its findings. The randomized controlled design, including both placebo and control groups, represents a key strength because many previous studies evaluating TENS for primary dysmenorrhea lacked sham conditions, limiting causal interpretation. The 3‐arm design employed in this study allows differentiation between true intervention effects, placebo responses, and natural symptom variation. In addition, baseline differences were controlled using ANCOVA, which strengthened internal validity by adjusting for preintervention pain and symptom levels. The use of both pain intensity (VAS) and menstrual symptom burden (DMSR) as outcome measures provided a comprehensive evaluation of the effects of TENS beyond pain alone. Intervention procedures were standardized, with predefined stimulation parameters, individualized electrode placement based on the primary pain location, and consistent session timing and duration. Support from a licensed physiotherapist in selecting and verifying the stimulation protocol further enhanced the reliability and safety of the intervention. Exclusion of participants who used analgesic medication during the intervention period also improved the clarity of the observed intervention effects.

Despite these strengths, certain limitations should be acknowledged. Anonymization was not feasible for participants in the control group because no device was applied, which may have influenced expectancy effects; however, the inclusion of a placebo group partially mitigates this limitation by allowing differentiation between placebo‐related and intervention‐specific outcomes. The study was conducted in a single setting among women residing in a public dormitory, which may limit generalizability to broader populations; nevertheless, participants were approached as women with primary dysmenorrhea rather than based on student status, and the underlying physiologic mechanisms of dysmenorrhea are unlikely to differ substantially across similar age groups. Additionally, outcomes were assessed within a single menstrual cycle, and long‐term effects of repeated TENS use across multiple cycles were not examined. Finally, stimulation intensity was adjusted individually to a strong but comfortable level rather than being fixed at a uniform electrical output. Although this approach reflects real‐world clinical practice, it may introduce some variability in provided stimulation intensity. The success of participant anonymization was not formally assessed in this study. Despite these limitations, the internal validity of the study is strengthened by its randomized, placebo‐controlled design.

## CONCLUSION

This randomized, single‐masked study provides evidence that TENS is an effective nonpharmacological intervention for reducing pain intensity in women with primary dysmenorrhea. Compared with both placebo and control conditions, TENS resulted in significantly lower postintervention pain scores with a large effect size, indicating a clinically meaningful analgesic benefit. In addition, TENS was associated with a reduction in overall menstrual symptom burden compared with the control group, suggesting that its effects extend beyond pain relief alone. The inclusion of both placebo and control groups strengthens the interpretation of these findings by distinguishing true intervention effects from placebo responses and natural symptom fluctuations. The results highlight the importance of placebo‐controlled designs in dysmenorrhea research and support the role of TENS as a safe, accessible, and noninvasive option for short‐term pain management during periods of peak menstrual discomfort. Given the limitations of pharmacologic treatments and the growing interest in nonpharmacological approaches, TENS may represent a valuable component of comprehensive dysmenorrhea management strategies. Future studies are warranted to explore the long‐term effects of repeated TENS use across multiple menstrual cycles and to evaluate its integration into routine nursing, midwifery, and women's health care practices.

## CONFLICT OF INTEREST

The author declares no conflicts of interest.

## Supporting information




**Appendix S1**. CONSORT 2010 Checklist of Information to Include When Reporting a Randomised Trial
